# Neural Differentiation of Induced Pluripotent Stem Cells for a Xenogeneic Material-Free 3D Neurological Disease Model Neurulation from Pluripotent Cells Using a Human Hydrogel

**DOI:** 10.3390/cimb45060290

**Published:** 2023-05-25

**Authors:** Luis Sebastian Alexis Valerio, Frederick Robert Carrick, Lina Bedoya, Sandeep Sreerama, Kiminobu Sugaya

**Affiliations:** 1 Burnett School of Biomedical Sciences, College of Medicine, University of Central Florida, Orlando, FL 32827, USA; valeriohlsa@hms.harvard.edu (L.S.A.V.); linasofiabema@knights.ucf.edu (L.B.); sandeepsreerama@knights.ucf.edu (S.S.); 2 Institute for Scientific Research and Technology Services (INDICASAT), City of Knowledge 0801, Panama; 3 Department of Biotechnology, Acharya Nagarjuna University, Guntur 522510, India; 4 MGH Institute of Health Professions, Boston, MA 02129, USA; 5 Centre for Mental Health Research in Association, University of Cambridge, Cambridge CB2 1TN, UK; 6 Department of Neurology, Carrick Institute, Cape Canaveral, FL 32920, USA

**Keywords:** iPS cells, neural stem cells, neurulation, extracellular matrix, platelets, Alzheimer’s disease

## Abstract

**Simple Summary:**

Alzheimer’s Disease (AD) is a leading cause of death and disability worldwide. Unfortunately, medical researchers have struggled to develop methods to understand why some people develop AD while others do not while also focusing on the disease’s late-stage consequences. We wanted to address this problem by modeling the disease using human-like physiological conditions. We wanted to explore the properties of human neural stem cells (NSCs) because these cells develop and maintain brain tissue. Conventional xenogeneic cultures cannot accurately recapitulate AD in vitro, and we needed a new model that would better represent the human brain’s structure, function, and pathology to study AD more effectively. We built a novel model using human induced pluripotent stem (iPS) cells combined with an extracellular scaffold of protein derived from human products and compared its basic stem cell properties versus the standard mouse-derived scaffold. We found different stemness and differentiation properties from cell lines cultured on the scaffolds, with more human-like characteristics found when using the human-derived scaffold. Our xeno-free model more closely mimics the human brain microenvironment, potentially increasing the quality of AD research. This is a technological advancement that the field can use in research experiments to understand AD better and to more effectively develop and test drugs and therapies to prevent and manage this terrible disease.

**Abstract:**

Alzheimer’s Disease (AD) is characterized by synapse and neuronal loss and the accumulation of neurofibrillary tangles and Amyloid β plaques. Despite significant research efforts to understand the late stages of the disease, its etiology remains largely unknown. This is in part because of the imprecise AD models in current use. In addition, little attention has been paid to neural stem cells (NSC), which are the cells responsible for the development and maintenance of brain tissue during an individual’s lifespan. Thus, an in vitro 3D human brain tissue model using induced pluripotent stem (iPS) cell-derived neural cells in human physiological conditions may be an excellent alternative to standard models to investigate AD pathology. Following the differentiation process mimicking development, iPS cells can be turned into NSCs and, ultimately, neural cells. During differentiation, the traditionally used xenogeneic products may alter the cells’ physiology and prevent accurate disease pathology modeling. Hence, establishing a xenogeneic material-free cell culture and differentiation protocol is essential. This study investigated the differentiation of iPS cells to neural cells using a novel extracellular matrix derived from human platelet lysates (PL Matrix). We compared the stemness properties and differentiation efficacies of iPS cells in a PL matrix against those in a conventional 3D scaffold made of an oncogenic murine-matrix. Using well-defined conditions without xenogeneic material, we successfully expanded and differentiated iPS cells into NSCs via dual-SMAD inhibition, which regulates the BMP and TGF signaling cascades in a manner closer to human conditions. This in vitro, 3D, xenogeneic-free scaffold will enhance the quality of disease modeling for neurodegenerative disease research, and the knowledge produced could be used in developing more effective translational medicine.

## 1. Introduction

Alzheimer’s Disease (AD), characterized by a loss of neurons and synapses, is a leading cause of death and disability worldwide [1,2,3]. Despite extensive research, little is known about how the AD microenvironment affects NSCs. Still, it is known that changes in extracellular matrix (ECM) dynamics and the abnormal accumulation of Tau neurofibrillary tangles and Amyloid β (Aβ) plaques are the hallmarks of the disease [1,2,3,4,5]. Previous research has widely focused on Aβ plaques and Tau tangles as the target for therapies, assuming that these features are the leading cause of AD. In contrast, our previous studies have reported that gliosis, another pathological factor of AD, is caused by the overexpression of the Aβ precursor protein (APP) independent of Aβ plaques, which prevents the neuronal differentiation of neural stem cells (NSCs) [6,7,8,9,10]. Therefore, developing an accurate in vitro model of the AD brain is imperative to study the effects of AD pathology on NSC biology. In such a precise model, patient-derived NSCs, neurons, and glia should co-exist and grow in an appropriate, physiologically compatible ECM. 

For this purpose, induced pluripotent stem (iPS) cells may be the ideal cellular component of the model because of their unique ability to differentiate into any cell type. We can induce pluripotency in somatic cells isolated from AD patients, differentiate them into NSCs, and further specialize them into neural cells. Neural cells are generated from neuroepithelial cells lining the neural tube. This developmental process, known as neurulation, is regulated by bone morphogenic proteins (BMP), transforming growth factors (TGFs), retinoic acid (RA), and sonic hedgehog (SHH) [11,12,13]. Several protocols have been established to differentiate induced pluripotent stem (iPS) and embryonic stem (ES) cells into NSCs. A combination of small molecules and recombinant proteins of the signaling cascade, such as dorsomorphin, SB431542, Noggin, and RA, have been used to control this differentiation process [11,12,13,14,15,16,17,18]. Of all the protocols developed thus far, the dual-SMAD inhibition via BMP and TGF signal regulation has shown the highest efficiency in yielding NSCs from iPS cells (iPSC-NSCs). However, the success of this process is highly dependent on the cell lines [18] and the culture conditions [19]. Unfortunately, the translation of this technology to clinical settings has been limited by using xenogeneic basal media and substrates based on animal products. 

Despite this, optimizing substrates free of xenogeneic products have yet to be broadly studied. Though xenogeneic, Matrigel is preferred over traditional substrates because it better mimics the microenvironment of the cells, facilitating molecular and cellular dynamics and cell-to-cell communication. Indeed, Matrigel is currently the standard substrate for pluripotent cells. However, Matrigel may not be adequate for physiological studies because it comprises vaguely defined variable factors and adhesion molecules (i.e., TGF, VEGF, FGF Collagen IV, and laminins different from humans). In addition, this scaffold is extracted from Engelbreth–Holm–Swarm mouse sarcomas, highlighting its malignant origin [20,21,22,23]. 

Single recombinant adhesion molecules are alternative candidates to replace the xenogeneic use of ECM [22,23,24]. Although they are defined with desirable content, these molecules fail to model the tridimensional structure of tissue during development and may lead to PS cells skipping important events that initiate diseases. Recombinant adhesive molecules may be desirable for cell cultures employed in cell therapy or isolating pure cell products. However, using only one type of molecule limits the dynamic events essential for cellular development and disease. Some ECM alternatives that provide a 3D environment have similar limitations, such as those composed of hyaluronic acid hydrogels [25] and fibrin hydrogels [26], because this involves single molecules.

In contrast, pluripotent stem (PS) cell-derived acellular matrices [27] or human fibroblasts [28] are desirable since they recapitulate tridimensional structures and represent a complex microenvironment. However, the production of these types of substrates is cumbersome. Considering the limitations of various xenogeneic and single-molecule substrates, a quickly produced ECM of human origin is needed in order to generate a human disease model in vitro. 

To this end, the field has considered xenogeneic-free cell culture media. Many commercially available alternatives for xeno-free basal media and human-derived supplements allow for the safer culturing of stem cells for clinical applications [24,29,30]. As a supplement, human platelet lysates (PL) are of particular interest because they have shown promising results in adult stem cell cultures (i.e., mesenchymal stem cells) and cell transplantation therapies [31,32,33]. The advantage of using platelets is that they are abundant in the blood, and their extraction is minimally invasive and requires minimal ethical concerns. PL has shown properties that promote angiogenesis, proliferation, neurogenesis, and neuroprotection in animal models because of its rich composition in PDGF, BMP, IGF-1, BDNF Vitronectin, and fibronectins [34,35,36,37].

Additionally, other plasma components, such as the cytoplasmic fragments of megakaryocytes, contain growth factors, adhesion molecules, and cytokines that are beneficial because they help heal [34,35]. Interestingly, the preparation of human PL lyophilized powder conserves the regenerative properties found in liquid extracts when applied to corneal epithelial cell lines in vitro [38]. Thus, human PL, or its product derivatives, inherently contains balanced and desirable regenerative factors needed for safer cell culture methodologies.

In the present study, we hypothesized that human PL-derived ECM allows differentiation of iPSCs into NSC, recapitulating their dynamic response to the AD microenvironment under xeno-free conditions. To this end, we used a commercially available PL-derived ECM, PL Matrix, to replace the traditional xenogeneic Matrigel. Combined with dual SMAD inhibition, the PL matrix may develop safe and high-quality human physiological microenvironments to produce neural progenitors in tridimensional cultures. Increased availability of cell cultures that closely simulate the in vivo conditions will enhance the development of neurodegenerative therapies and improve current personalized medicine models.

## 2. Materials and Methods

### 2.1. iPS Cell Cultures

The iPS cell lines used from Cellular Dynamics (Madison, WI, USA) were CW50018 (RRID:CVCL_DK51) derived from a 58-year-old Caucasian AD female patient and CW50064 (RRID:CVCL_DK85) as noncognitive decline control cells from an 80-year-old Caucasian male. Supplementary Figure S1 confirms that the acquired iPS cells possess biomarkers and functional pluripotency properties. To verify the biomarkers, we used the human pluripotent stem cell marker antibody panel plus (R&D systems, catalog #SC009, Minneapolis, MN, USA), according to manufacturer’s instructions. To test the differentiation into the three germ layers, the human pluripotent stem cell functional identification kit (R&D, catalog #SC027B) was used, according to manufacturer’s instructions. The iPS cell lines were expanded on 6-well tissue culture plates using mTeSR media (Stem cells technologies, Cambridge, MA, USA) enriched with ROCK inhibitor (H1154 10 ug/mL, R&D Systems) until full confluency was reached. The media were changed daily, and cells were passaged using Versene (Invitrogen, Waltham, MA, USA) as a detachment factor. The substrates used for expansion were commercially available hydrogels from acellular ECMs: PL Matrix (PL BioScience GmbH, Aachen, Germany, Patent EP2723850B1) and Matrigel (Corning, NY, USA), both used according to the manufacturer’s instructions. 

### 2.2. Neural Induction

The general procedure for the neurulation procedure is shown in Supplementary Figure S2. We followed the dual-SMAD inhibition method with minimum modifications for neurulation using PL Matrix (PL BioScience GmbH, Aachen, Germany) and Matrigel (Corning) as a coat for cell adhesion. On days 1–5, iPS cells were maintained daily with neurulation media containing DMEM:F12 (Invitrogen), 10% KSR (Invitrogen), 5% Glutamax (Invitrogen), 5% NNEA (Invitrogen), and 1% antibiotics and antimycotics (Invitrogen), supplemented with TGF inhibitor (10uM SB431542, R&D Systems) and BMP inhibitor (500 ng/mL Noggin, R&D Systems). On days 5–7, cells were exposed to 75% of the neurulation media without TGF inhibitor but retaining Noggin and 25% of the N2 media containing neurobasal media (Invitrogen) with 1X N2 supplement (Invitrogen). On days 7–9, the cells were exposed to 50% neurulation media without TGF inhibitor and 50% N2 media but retaining Noggin. On days 9–11, cells were exposed to 25% neurulation media without TGF inhibitor, retaining Noggin and using 75% N2 media. From day 11 onward, the expected NSCs were maintained and expanded for further experimentation or cryopreservation using media containing neurobasal media (Invitrogen), 1XN2 supplement (Invitrogen), 1XB27 (1:50, Invitrogen), basic Fibroblast Growth Factor (bFGF 20 ng/mL, R&D Systems), Epidermal Growth Factor (rhEGF 20 ng/mL, R&D Systems), Heparin (0.5 U/mL, Sigma, St. Louis, MO, USA), and 1% antibiotics Penicillin/Streptomycin (Invitrogen). The EGF and bFGF were added only at 100% N2 media after day 11 because they are used to maintain and expand the expected NSCs in their stemness mode. The EGF and FGF were not used during the gradient of media because, otherwise, the differentiation of iPS cells into NSCs might not be that robust for stemness signaling.

### 2.3. Neural Stem Cell Expansion and Differentiation

After day 12, the iPSC-NSC lines CW50018 and CW50064 (Cellular dynamics) in the form of neurospheres or adhered cells were detached using Versene (Invitrogen), dissociated using a syringe and needle, and transferred into T75 suspension flasks containing expansion media, composed of DMEM F:12, B27 (1:50, Invitrogen), and 1% antibiotics (Invitrogen). For analysis, three 8-well chamber slides or three 12-well tissue culture plates were coated with PL Matrix (PL BioScience GmbH, Aachen, Germany) or Matrigel (Corning) before seeding the stem cells. After reaching confluency, cells were fixed with 4% paraformaldehyde (PFA, Sigma) and preserved on cold PBS 1X (Sigma) for the stem cell markers immunocytochemistry. 

Another batch of iPSC-NSCs was seeded on three 8-well chamber slides or three 12-well tissue culture plates coated with PL Matrix or Matrigel for differentiation. Cells were cultured, refreshing the differentiation media, composed of neurobasal media (Invitrogen), N2 supplement (Invitrogen), and brain-derived neurotrophic factor (BDNF 100 ng/mL, R&D Systems), each third day for two weeks. Other differentiating factors and their variable concentration coming from the composition of the addressed scaffolds were expected to provide variability of differentiated cells. Twenty-one days of differentiated culture cells were fixed using 4% PFA (Sigma) for further analysis.

### 2.4. Immunocytochemistry

After fixation using 4% PFA (Sigma), samples were washed three times using PBS 1X (Sigma) with 1% BSA (Sigma). Then, samples were permeabilized using 0.3% Triton-X (Fisher Scientific, Waltham, MA, USA) and 10% normal donkey serum (Jackson ImmunoResearch, West Grove, PA, USA) for 1 h. After this, samples were washed four times with PBS 1X (Sigma) and incubated with the primary antibodies against GFAP (1:1000, Invitrogen), βIII tubulin (1:1000 Invitrogen), Sox1 (1:1000 Invitrogen), Musashi1 (1:1000, Invitrogen), Nestin (1:1000, Chemicon) or HuNu (1:1000 Chemicon) overnight at 4 °C. The next day, samples were incubated with 0.1% BSA/PBS (Sigma) and secondary antibodies (1:500) conjugated with FITC or TRITC for two hours at room temperature. Samples with the secondary antibody were washed three times using PBS 1X and mounted on a well or microscope slide with a water-based mounting solution containing DAPI (Vector Laboratories, Newark, CA, USA). For imaging, we used the Zeiss 719 confocal microscope and the Zeiss AxioObserver microscope and objectives for 10× and 20× magnification. We used the ImageJ server to extract immunostaining intensities. 

### 2.5. Survival Assays

The cell survival assay of the iPS cell lines was subcultured at a density of 25,000 cells using tissue culture plastic plates coated with PL Matrix and Matrigel. The experiments were conducted three independent times with three replicates each, n = 3. The cells were incubated at 37 ℃ with 5% CO_2_ for five days with daily media changes with mTeSR (Stem Cell Technologies, Vancouver, BC, USA). Then, the cells were stained with Calcein/Propidium Iodide (Invitrogen). Similarly, the differentiated NSCs from the iPS cell lines were tested for survival. However, the cell culture medium was used to expand NSC cells as described above. In all cases, three fluorescence images and phase-contrast photomicrographs were taken for each culture. Then, manual counting of death and live cells was conducted using the server ImageJ and expressed as a percent of live cells (stained on green). Then, we used PRISM 9.5.1 for the statistical analysis and graphics creation. 

### 2.6. Quantification of Fluorescence Intensities

For the quantification of the fluorescence intensities of the immunostained samples, we used the software ImageJ (NIH). We generated the graphic using the software PRISM. We randomly took images in triplicates for each of the conditions addressed in this study. The images were converted into an 8-bit digital scale, and a threshold of regions of interest (ROI) for all the cells from the images was set to obtain fluorescence intensities. Their negative images were taken to subtract the background signal intensities of the immunostaining samples. Then, we normalized the data using the fluorescence intensities from DAPI signaling and expressed the data as a ratio of fluorescence intensities from the average measured ROI normalized to DAPI.

### 2.7. Statistics

We used the PRISM software (version 7.0e) to analyze statistical differences using the two-way ANOVA for multiple comparison groups or *t*-student tests to compare two groups of the genes and antigens studied accordingly. With this server, we created the graphics shown in the present study. Differences of *p* < 0.05 were considered significant. 

## 3. Results

### 3.1. The Platelet-Derived Extracellular Matrix Allows the In Vitro Culture of Human iPS Cells 

**Figure 1:**
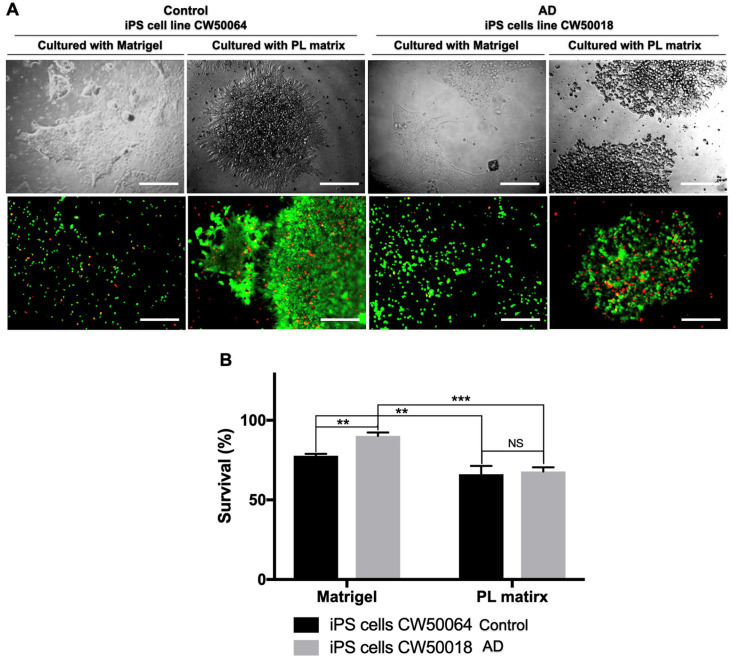
Comparison of hiPS cells’ survival seed on PL Matrix and Matrigel after five days using calcein and propidium iodide. The cell lines CW50064 and CW50018 have a percentage of survival (calcein is green; PI is red) significantly higher when seeded on Matrigel versus PL Matrix (**A**,**B**). The PL matrix allows the cells to become recruited and quickly form a colony with sharp borders and a 3D-like structure. Matrigel shows cells scattered individually or in clusters in a 2D manner. Both of the ECMs allowed the iPS cells to grow. ** *p* < 0.01, *** *p* < 0.005. Scale bar, 100 µm.

Figure 1 shows the in vitro cultures of human iPS cell lines CW50064 (control) and CW50018 (AD) using the biomimetic ECMs PL Matrix and Matrigel. In both ECMs, the human iPS cell lines exhibit tightly compact, sharp-edge colonies with rounded cells with prominent nuclei. Moreover, while human iPS cell line colonies cultured on Matrigel show a flat, two-dimensional colony structure, the settlements grown on PL Matrix display a tridimensional block-like structure. Neither of the substrates reveal any cluster that resembles an embryoid body. After five days, we found no difference in the cell survival rates between the control (CW50064) and AD (CW50018) cell lines cultured on the PL matrix (*p* = 0.8937). However, the cell survival rates between both lines differed when Matrigel was used (*p* = 0.0058). Additionally, when cultured on Matrigel versus PL matrix, there is a significant difference in the survival rates for both human iPS cell lines, CW50064 (*p* = 0.0087) and CW50018 (*p* = 0.0001). These data suggest that human-derived PL-ECM offers more stable cultures for the studied human iPS cells.

**Figure 2:**
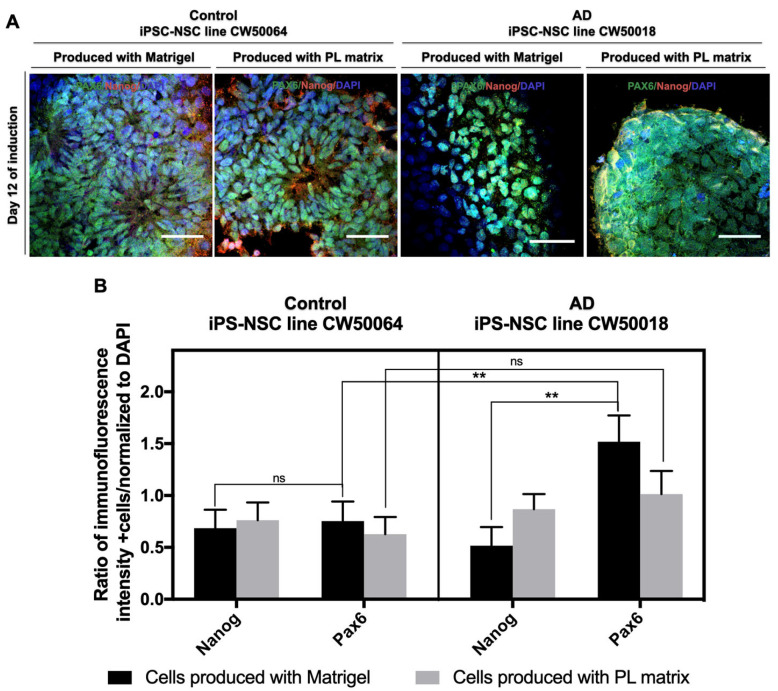
NSC differentiation from hiPS cells using PL Matrix and Matrigel via the dual SMAD inhibition method. After 12 days of neurulation, the hiPS cells CW50064 and CW50018 expressed NSC marker Pax6 (green) and stemness marker NANOG (red); however, they show differences in the morphology of the colonies formed. The cell line CW0050064 displays a neural rosette-like shape, while the line CW50018 shows clusters (**A**). There is not a significant difference in the NANOG protein expression levels in the iPSC-NSCs produced with PL matrix (**B**). Nuclei were counterstained with DAPI ** *p* < 0.01. Scale bar, 25 µm.

In addition to the biomimetic ECMs PL Matrix and Matrigel, we recruited a dual-SMAD inhibition method to produce iPSC-NSCs under serum-free conditions. Small molecules, namely, Noggin and SB431542, were used in the process (Supplementary Figure S2). As shown in Figure 2, after 12 days of neurulation, the human iPS cells CW50064 and CW50018 expressed NSC marker Pax6 and stemness marker NANOG when cultured on either ECM. Pax6 protein expressions were significantly higher in CW50018 than in CW50064 (*p* = 0.0046) on cultures using Matrigel. However, we did not observe a difference in NANOG protein expression between these cell lines, regardless of ECM choice. By comparing the PL matrix and Matrigel, there is no significant difference in NANOG protein expression levels between the control (CW50064) (*p* = 0.9998) and AD (CW50018) iPSC NSCs (*p* = 0.8957).

Interestingly, Matrigel-raised cultures show a significant difference (*p* = 0.0011) in their NANOG protein expression levels compared to the PAX6 in AD (CW50018) but not in the control (CW50064) (*p* > 0.9999). This difference was not detected when the cultures were grown using a PL matrix in the AD cell line (*p* = 0.9791) or the control cell line (*p* = 0.9926). PAX6 protein expression levels are significantly higher (*p* = 0.0046) for cultures raised in Matrigel. In cultures raised in the PL matrix, the PAX6 protein expression levels are higher for the AD cell line (CW50018) than for the control cell line (CW50064), but the levels are not significantly different (*p* = 0.2919). On the other hand, these cell lines exhibit differences in the morphology of the colonies formed. While the control cell line (CW50064) displays a neural rosette-like shape, the AD cell line (CW50018) is sphere-like, and this may be attributed to the nature of each of the cell lines and not the matrices. These data suggest there may be inherent differences between the cell lines because of their unique physiology, accounting for differences in treatment response. However, this is not correlated to the extracellular matrix. Thus, the PL matrix is suitable for the culture of iPS cells and their differentiation towards an NSC lineage. 

### 3.2. Expansion and Characterization of iPSC-NSCs Produced Using Platelet-Derived Extracellular Matrix

**Figure 3:**
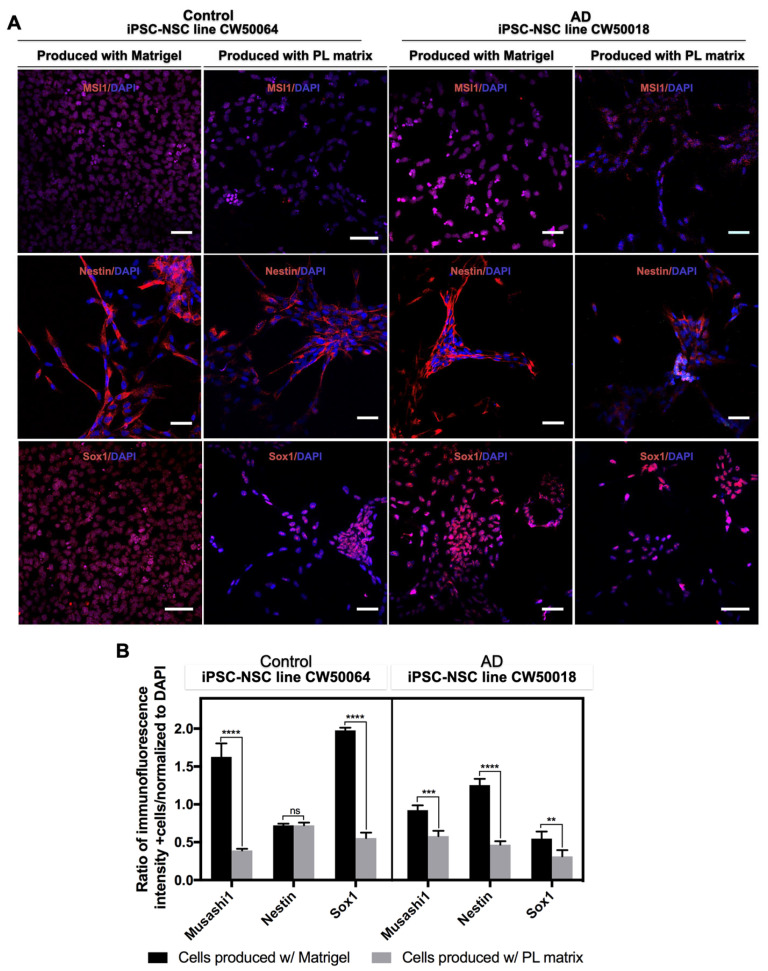
Characterization of iPSC-NSCs produced with PL matrix and Matrigel. When made using PL matrix and Matrigel, the hiPS cells CW50064 and CW50018 express NSC markers MSI1, Nestin, and Sox1 (**A**,**B**). Nuclei were counterstained with DAPI. The protein expression levels show that the iPSC-NSCs lines behave differently when cultured on both ECM; however, their relative pattern is conserved on both lines. ** *p* < 0.01, *** *p* < 0.005, **** *p* < 0.0001. Scale bar, 100 µm.

On day 12 of differentiation, the control (CW50064) and AD (CW50018) iPSC-NSC cultures were dissociated and transferred into a suspension flask using serum-free media. The cells grew and formed neurospheres, which were passaged through mechanical disassociation of the spheres with a surgical blade, syringe, and needle. As shown in Figure 3, the human iPSC-NSCs lines, both control (CW50064) and AD (CW50018), produced on PL Matrix vs. Matrigel, express NSC markers MSI1, Nestin, and Sox1. Both cell lines have high expression levels for NSC markers on Matrigel, except for Nestin in the control cell line (CW50064). Precisely, for the iPSC-NSCs control cell line (CW50064), there is a significant difference in the protein expression levels of MSI1 (*p* < 0.0001) and Sox1 (*p* < 0.0001), whether cultured on Matrigel or PL Matrix. On the other hand, the iPSC-NSC AD cell line (CW50018) shows a notable difference in the protein expression levels of Nestin (*p* < 0.0001), in addition to the differences in the expression levels of MSI1(*p* = 0.0001) and Sox1 (*p* < 0.0066) when cultured on Matrigel vs. the PL matrix. The differences in NSC marker expression levels may be attributed to the variable physiology of the cell lines responding to the different substrates. 

**Figure 4:**
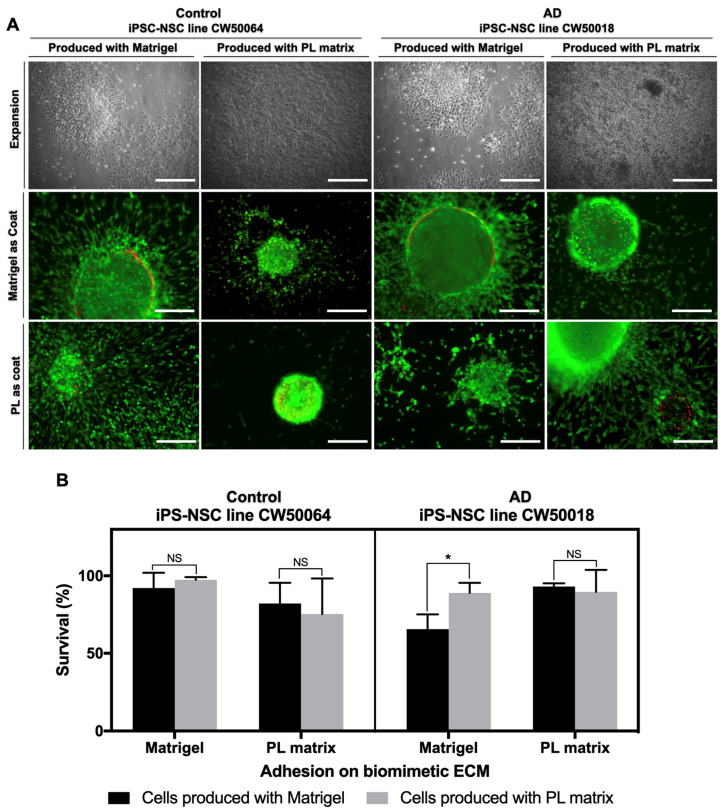
Compatibility of hydrogels between iPSC-NSCs lines CW50064 and CW50018. The generated iPSC-NSCs raised on the PL matrix can grow on Matrigel as a medium (**A**,**B**). Similarly, the ones presented on Matrigel can grow on the PL matrix. Staining was performed using calcein (shown in green color) and propidium iodide (Shown in red color). * *p* < 0.05. Scale bar, 100 µm.

Next, we evaluated the cell survival response of the two control (CW50064) iPSC-NSC lines and the two AD (CW50018) iPSC-NSC lines initially grown and produced using PL Matrix and Matrigel. We checked survival using coats from their original source and compared them with their counterpart matrix to assess their survival efficacy in a new culture condition. As shown in Figure 4, cellular adhesion on the biometric ECMs, 48 h after seeding, allowed for the survival of the iPSC-NSCs of both cell lines. There is a small but significant difference in the iPSC-NSC AD cell line (CW50018) produced and later cultured on Matrigel versus those made with Matrigel but subsequently cultured on PL Matrix (*p* = 0.0369). Otherwise, the viability pattern is conserved between the iPSC-NSC lines produced in one biometric ECM substrate and cultured using the other. Therefore, it can be inferred that the cells are compatible with substrates and culture conditions different from those used for their original production. Altogether, these data suggest that, similarly to Matrigel, the composition of the PL matrix allows for producing iPSC-NSCs with a conserved pattern of protein expression levels and culture compatibility between different biomimetic ECMs.

### 3.3. In Vitro Differentiation of the iPSC-NSCs Produced Using Platelet-Derived Extracellular Matrix

**Figure 5:**
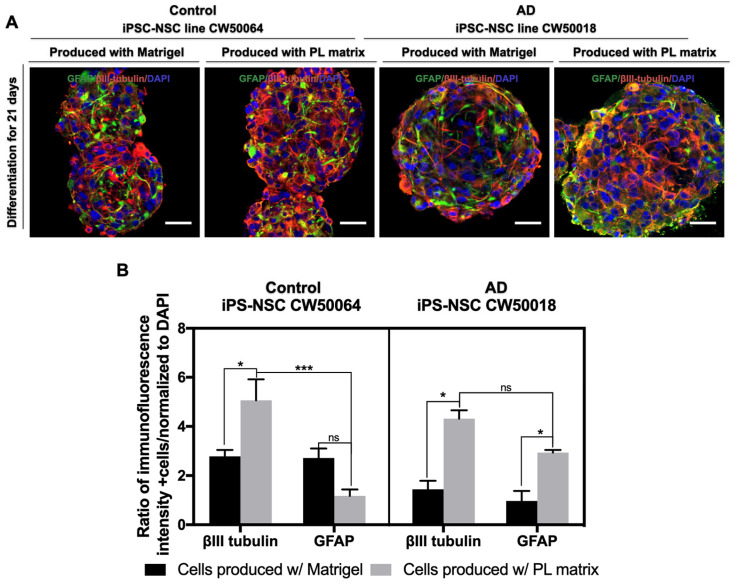
Differentiation of iPSC-NSCs lines CW50064 and CW50018. Nuclei were counterstained with DAPI. Cells yielded positive immunostaining for βIII-tubulin (red) and GFAP (green) and formed web-like structures inside the spheres (**A**). The ratio of fluorescence intensities related to the protein expression levels shows that the iPSC-NSCs lines have different behavior when differentiated on cultures with other ECM (**B**), according to their physiological nature. * *p* < 0.05, *** *p* < 0.005. Scale bar, 25 µm.

Next, we tested the ability of the iPSC-NSCs from the control (CW50064) and AD (CW50018) cell lines to undergo neural differentiation using serum-free basal media. As shown in Figure 5, after 21 days, the cells cultured on their respective biomimetic ECMs yielded positive immunostaining for βIII-tubulin and GFAP. The cells formed a web-like network of neuronal and glial cells within the 3D spheres. Cells raised on Matrigel and PL Matrix show significant differences in the expression of neural differentiation maker βIII-tubulin for both control (CW50064) (*p* = 0.0184) and AD (CW50018) (*p* = 0.0039) cell lines. No significant differences were found in the GFAP expression for the control (CW50064) cell line cultured on Matrigel and PL Matrix. However, there was a significant difference in GFAP (*p* = 0.0451) expression between Matrigel and PL Matrix for the AD (CW50018) cell line. 

When we compared the protein expression levels of neuronal (βIII-tubulin) and glial (GFAP) markers for the control cell line (CW50064), there was a significant difference (*p* = 0.0005) for the PL matrix cultures but not for the Matrigel cultures. No significant differences were found in the protein expression levels of neuronal and glial markers for the AD cell line (CW50018) for the PL matrix or Matrigel cultures. These data suggest that the resultant iPSC-NSCs produced from control (CW50064) and AD (CW50018) cell lines, irrespective of whether Matrigel or PL Matrix was used, possess a multipotency that can produce neurons and glia according to their physiological nature.

## 4. Discussion

This study reports that a commercially available human platelet-derived ECM (PL Matrix) is suitable to culture human iPS cells from AD (CW50018) and control (CW50064) cells and allows driving iPS cell differentiation towards NSCs in a xeno-free manner, as shown in Figure 1 and Figure 2. The NSCs produced in the cultures supported by this type of human-derived ECM can be expanded and differentiate into neurons and glia, as shown in the Figure 3. The uses of iPS cell cultures with an environment free of xenogeneic material is needed to establish human-compatible culture conditions [29]. This technical approach may generate more human like-physiological conditions for in vitro disease models meaningful for future personalized medicine or even safer cell therapies. The ongoing research is delimitated to studying neural stem cell production from iPS cells and evaluating their properties because these cells are key players in the maintenance of the adult brain microenvironment and are responsible for its development during embryogenesis. To this end, we acquired commercially available iPS cell lines with their biomarkers and functional pluripotency verified (shown in Supplementary Figure S1). In the pluripotency state, the cells may not show physiological or metabolic activity. Further studies will be needed to study what happens to the physiological properties for neuronal Alzheimer disease cell lines on long-term culture experiments using the conditions established herein. 

In the current study, we tested the potential uses of ECMs from human platelet lysates, commercially available as PL Matrix, to culture iPS cells and compared their similarities with cultures grown on Matrigel. PL Matrix and its counterpart, Matrigel, contain different amounts and types of growth factors and adhesion molecules that may allow human iPS cell lines, i.e., AD (CW50018) and control (CW50064), to react differently for survival and differentiation (as shown in Figure 1 and Figure 2). The varied compositions of the two matrices can contribute to the cell lines’ variance in adherence, growth, differentiation to NSCs, and, finally, terminal differentiation into neurons and glial cells. In addition to the physiological nature and age of the donors of the iPS cell lines used, the role of the biomaterial used on the cultures in the production of NSCs is addressed. It has been shown that different iPS cell lines possess different innate differentiation responses to the ECM. These specific characteristics produce NSCs with discrete properties and behaviors [15], depending on the various biomaterials that compose the scaffold. Many biomaterials have been optimized to standardize cell culture techniques. One method is creating synthetic hydrogels, such as gelatin methacrylate or hyaluronic acid, which provide iPSC-NSC cultures with the ability to differentiate into functional neurons [26,39]. However, this method demands complex procedures requiring cell encapsulation into the gel, which is costly and inefficient. The alternative to synthetic hydrogels is recombinant adhesive proteins, such as Cadherins, laminins, and Vitronectin, present in the ECM [22,23,24]. However, these biomolecular coatings do not mimic the complex tridimensional environment of tissues. A better approach would be to use decellularized ECM. The behavior of iPS cells and iPSC-NSCs from several ECMs prepared in different ways has been previously investigated. One example is the decellularized ECM derived directly from embryoid bodies, with or without differentiated cells, produced from iPS cells [20]; however, this method requires long-term cultures to make said biomaterials. Other examples for culturing iPSC-NSCs are the ECM from porcine central nervous systems [40,41] and the traditional hydrogel-based ECM derived from decellularized mice sarcomas, Matrigel, used in the present study [42].

Nevertheless, the xenogeneic nature of these techniques may fail to support the process that occurs in human development, which is essential for disease modeling. Significantly, substrate stiffness, composition, and topography affect the physiology and behavior of iPS cells, affecting their differentiation [43,44,45]. Dynamic changes such as the matrix and the inconsistencies in its composition may limit their use in personalized clinical therapy. Additionally, the animal-derived cancer-tissue origin of some matrices, such as Matrigel, can compromise their safe use in translational medicine. 

Matrigel has become the standard substrate and a suitable scaffold for culturing iPS cells that differentiate into specialized cells for 2D and 3D cultures. In contrast with Figure 1 of the current study, Matrigel and the novel PL Matrix allow for the culture of pluripotent cells in a tridimensional manner, with high survival rates. It is important to highlight that 3D cultures influence cells’ bioprocesses and transcriptional profiles, an important characteristic that may be lost in 2D cultures [43]. In our study, we found that neurulation can co-occur in both Matrigel and PL Matrix, and the resultant cells yielded PAX6-positive immunostaining and showed neural rosette-like structures resembling the neural tubes and spheres. Although, the pattern of NANOG expression levels is not conserved between the two cell lines when grown on different scaffolds, these data suggest the possibility of heterogeneous cultures of different qualities of neural progenitors. Interestingly, we found lower NANOG expression levels and higher expression levels of PAX6 in Matrigel (*p* = 0.0011) and PL Matrix for the AD (CW50018) iPSC-NSCs. However, the NANOG and PAX6 expression levels are almost identical, with non-significant differences for the control (CW50064) iPSC-NSCs. In the same way, after expanding the iPSC-NSCs, cells displayed positive protein expression for NSC markers MSI1 and Sox1. For both cell lines, the expression pattern is maintained at significantly higher levels on Matrigel than on the PL matrix. The NSC marker Nestin displays a different pattern of expression levels in iPSC-NSCs produced in cultures with PL Matrix or Matrigel (control (CW50064) was non-significant, while AD (CW50018) was (*p* < 0.0001)). 

Although little is known about the genotypic influence on NSCs in Alzheimer’s Disease, the differences found in this study between the same cell line may be due to the natural composition of the scaffold. Each scaffold may have remodeled the ECM to create its microenvironment. It is known that TGF-β signaling has beneficial or deleterious roles in Aβ protein levels and a relationship to stemness. Matrigel is particularly rich in TGF and FGF compared to the PL matrix (which is richer in PDGF and IGF). The stemness factors expressed from the cell lines may dynamically interact with the scaffold microenvironment and behave differently according to physiology [34,35,36]. 

Intriguingly, the iPSC-NSCs from Alzheimer’s disease and the non-AD control cell line yielded positive immunostaining for βIII-tubulin and GFAP when cultured on Matrigel and PL Matrix. The cells formed a web-like network of neuronal and glial cells within the 3D spheres. There is a significant difference in the protein expression levels of neuronal differentiation marker βIII-tubulin between the control (CW50064) (*p* = 0.0184) and AD (CW50018) (*p* = 0.0039) iPSC-NSC cell lines cultured using either matrix. While the glial marker GFAP displays significant differences in the AD iPSC-NSCs (*p* = 0.0451), no significant differences were found in the iPSC-NSC controls using Matrigel or PL Matrix. The protein expression levels of neuronal and glial markers for the control (CW50064) cell line have significant differences (*p* = 0.0005) for the PL matrix but not Matrigel cultures.

On the other hand, the protein expression levels of neuronal and glial markers for the AD (CW50018) cell line have no significant differences for cultures grown in the PL matrix or Matrigel. In contrast to their original host from the different cell lines, these data suggest that the NSCs possess multipotency and can produce neurons and glial cells in vitro. The differentiation seems to be according to their physiological nature in the ECM. The higher levels of glial markers in the PL matrix-raised AD cells may be attributed to the response of the NSCs to the PDGF and BMP proteins known to regulate glial differentiation from NSCs. These proteins will likely be present dynamically in the PL matrix [9,10,34,35,36,37].

Similarly, growth factors such are BDNF and IGF-1, also abundant in platelet lysates, may influence the higher rates of neuronal markers in both the cell lines grown in the PL matrix. Therefore, balanced and human-compatible ECMs may recapitulate the neural stemness and differentiation events in the brain’s microenvironment. On the other hand, an imbalanced amount of growth factors, such as TGF, VEGF, FGF, and collagens from mice sarcomeres, are likely dynamically active on Matrigel. These factors may influence differentiation towards neurons and glia, recreating a microenvironment in a way that is different from human physiology. 

It has been indicated that when PL is used as serum replacement for media preparation, it facilitates the culture of stem cells [26,27,28]. Its composition is rich with growth factors PDGF, BDNF, BMP, IGF-I, VEGF, fibronectins, and vitronectin cell adhesion molecules [34,35,36,37]. The extracts from PL promote angiogenesis, proliferation, neurogenesis, and neuroprotection in animal models. A model of cell cultures with a genotypic predisposition to diseases may differ from non-disease cultures, which is highlighted by the use of different types of scaffolds to influence the properties and behavior of the cells. Thus, these data may suggest that the PL matrix recapitulates the developmental events needed to produce NSCs, neurons, and glial cells parallel to the human physiological microenvironment. This optimized version of the dual-SMAD inhibition method may develop safe and high-quality neural progenitors to accelerate the development of neuroregenerative therapies and improve current personalized medical models.

## 5. Conclusions

The scaffold in which iPS cells are grown and specialized is a crucial factor to consider when using iPS cell technology for disease modeling, drug screening, and therapy. Current alternatives for the growth and specialization of iPS cells into NSCs have yet to be further studied and evaluated because they fail to mimic the tissue environment of the human body. This study presented the human platelet lysate (PL) matrix as a safe, suitable, and high-quality 3D EMC alternative for the growth and specialization of AD iPS cell lines into NSCs based on the dual-SMAD inhibition method. Our results indicate that the neural progenitor cells created with this cell culture methodology may closely mimic the behavior of the human microenvironment and could represent a more suitable alternative to improve current neurodegenerative therapies for further development of personalized medical models for neurodegeneration research. 

## Data Availability

All data generated or analyzed during this study are included in this published article.

## Supplementary Materials

The following supporting information can be downloaded at: https://www.mdpi.com/article/10.3390/cimb45060290/s1, Figure S1: Characterization of iPS cell lines acquired from cellular dynamics, Figure S2: Representative scheme for the process of differenting iPS cells into neural cells.
