# Supplementary material for: Neural Differentiation of Induced Pluripotent Stem Cells for a Xenogeneic Material-Free 3D Neurological Disease Model Neurulation from Pluripotent Cells Using a Human Hydrogel
Source: Curr Issues Mol Biol. 2023 May 25;45(6):4574–88. doi: 10.3390/cimb45060290 (PMC10297326; doi:10.3390/cimb45060290)
Supplement: Supplementary file 1 [file cimb-45-00290-s001.zip › cimb-2390646-supplementary.pdf]

Supplementary Figures:

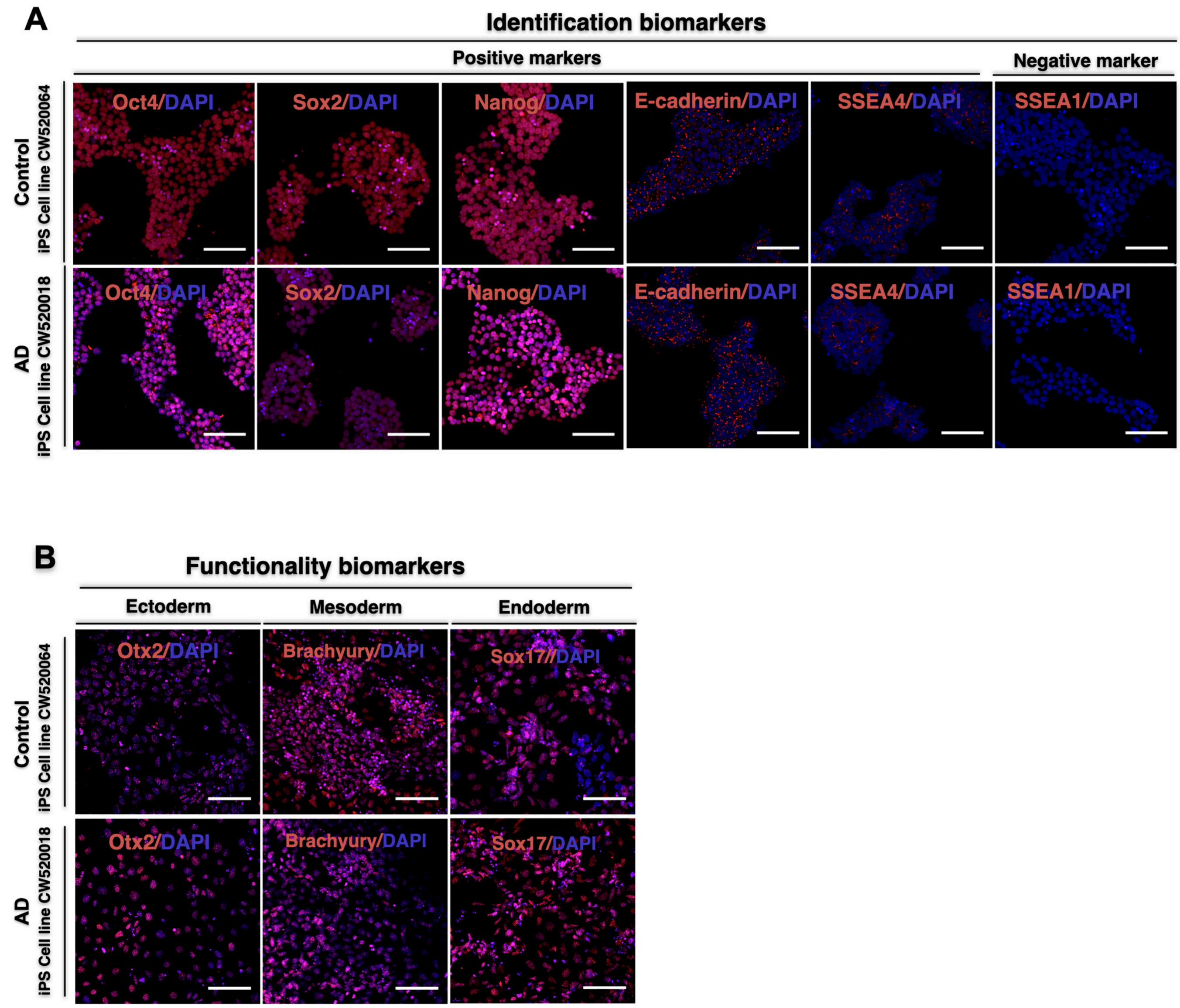


Supplementary Figure S1. Characterization of iPS cell lines acquired from cellular dynamics. The iPS cell line control CW50064 and the patient-specific Alzheimer disease (AD) CW50018 display typical morphologies of pluripotent cells and express Nanog, Oct4, Sox2, SSEA-4, E-Cadherine while negative for SSEA-1 (A). In addition, the cell lines display functional biomarkers Otx2, brachyury, and Sox17, typically expressed on ectoderm, mesoderm, and endoderm respectively (B). Scale bar, 100 μm.

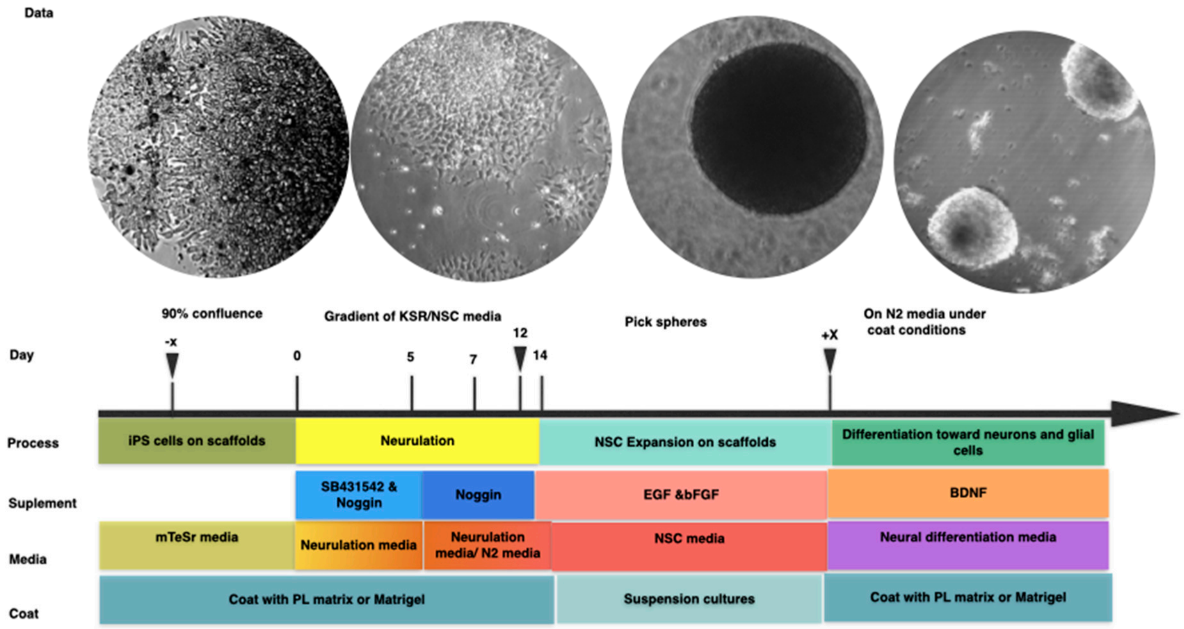


Supplementary Figure S2. Representative scheme for the process of differenting iPS cells into neural cells. The iPS cell lines were expanded using mTeSR media on contenders coated with either Matrigel or Pl matrix. Then, SMAD proteins were regulated by inhibition of BMP and TGF signaling using SB431532 or Noggin. On days 1-5, iPS cells were maintained daily with neurulation media supplemented with TGF inhibitor SB431543 and BMP inhibitor Noggin. On days 5-7, cells were exposed to 75% of neurulation media without TGF inhibitor and 25% of N2 media containing neurobasal media and N2 supplement, retaining Noggin. On days 7-9, the cells were exposed to 50% neurulation media without TGF inhibitor and 50% N2 media, but retaining Noggin as well. On days 9-11, cells were exposed to 25% of neurulation media without TGF inhibitor, retaining Noggin and using 75% of N2 media. From day 11 onward, the expected NSCs were maintained and expanded for further experimentation or cryopreservation using neural stem cell (NSC). Finally, the differentiation was done by using neural differentiation media supplemented with BDNF.
